# The Launch of an International Animal Papillomavirus Reference Center

**DOI:** 10.3390/v11010055

**Published:** 2019-01-14

**Authors:** Koenraad Van Doorslaer, Joakim Dillner

**Affiliations:** 1 School of Animal and Comparative Biomedical Sciences, The BIO5 Institute, Department of Immunobiology, Cancer Biology GIDP, Genetics GIDP, and UA Cancer Center, Tucson, AZ 85721, USA; 2 International HPV Reference Center, Department of Laboratory Medicine, Karolinska Institutet, 14186 Stockholm, Sweden; Joakim.Dillner@ki.se

The *Papillomaviridae* is a family of DNA viruses. These viruses are associated with a range of human and animal diseases. With currently 226 distinct characterized human papillomaviruses (HPVs), the majority of the established papillomavirus (PV) diversity is isolated from humans [1]. While such viral diversity has not yet been described for any non-human animal species, it is likely that increased sampling will likewise yield dozens of papillomaviruses on non-human hosts. Specifically, the use of high-throughput, unbiased screens of non-human “viromes” is expected to dramatically increase the number of non-human papillomaviruses in the (near) future. Animal papillomaviruses have played, and continue to play, critical roles in elucidating papillomavirus biology and HPV vaccine development.

Formal recognition of a putative human papillomavirus isolate requires that a full-length, cloned genome be submitted to the International Human Papillomavirus Reference Center (www.hpvcenter.se) [2]. The Reference Center confirms the viral sequence, assesses whether the isolate is novel, and assigns a formal type number to the isolate. Moreover, the Reference Center acts as a repository that provides these cloned genomes to the research community [3]. This endeavor has been very successful over the past 30 years [4].

To date, the situation for animal papillomaviruses has been very different. Lack of community support during the same period has made access to newly identified animal papillomavirus genomes significantly more bothersome. Additionally, the non-curated nomenclature of animal PVs has resulted in confusion and multiple uses of the same or similar abbreviations for different PVs. For example, the abbreviation ChPV1 has been used both to name a goat virus [5] and a chimpanzee virus [6].

The naming of different animal papillomaviruses follows a set of guidelines [7]. However, to avoid duplication of virus names and abbreviations, a coherent nomenclature system requires knowledge of all isolated viruses, regardless of their publication status. Therefore, we propose creating an International Reference Center for Animal PVs (www.animalPVs.org). This newly created center will be housed at the University of Arizona. Cloned viral genomes, associated sequence information, and metadata should be submitted to the Animal papillomavirus reference center. The submitted materials will then be analyzed by the International HPV Reference Center in the same manner as currently performed for human papillomaviruses. Following the analysis of submitted materials, the animal papillomavirus reference center will formally recommend names for the viral isolates. We are cognizant that many viral genomes are identified through high-throughput metagenomics, and cloning viral genomes may not always be feasible. In this case, the sample from which the novel virus was identified can be submitted as the “index sample” for independent verification of the sequence by the Reference Center.

We cannot require official designation as a requisite for publication, but we hope that authors, reviewers, and editors will join us in this effort. We believe that a robust and workable papillomavirus nomenclature is paramount to communication and reproducibility within the papillomavirus community, as well as for animal papillomaviruses.
